# Adverse Cardiovascular Outcomes associated with Coronary Artery Bypass Surgery and Percutaneous Coronary Intervention with Everolimus Eluting Stents: A Meta-Analysis

**DOI:** 10.1038/srep35869

**Published:** 2016-10-24

**Authors:** Pravesh Kumar Bundhun, Manish Pursun, Abhishek Rishikesh Teeluck, Akash Bhurtu, Mohammad Zafooruddin Sani Soogund, Wei-Qiang Huang

**Affiliations:** 1 Institute of Cardiovascular Diseases, the First Affiliated Hospital of Guangxi Medical University, Nanning, Guangxi, 530027, P. R. China; 2 Guangxi Medical University, Nanning, Guangxi, 530027, P. R. China

## Abstract

This study aimed to compare the mid-term adverse cardiovascular outcomes associated with Coronary Artery Bypass Surgery (CABG) and Percutaneous Coronary Intervention (PCI) with Everolimus Eluting Stents (EES). Electronic databases were searched for studies comparing the mid-term (>1 year) adverse cardiovascular outcomes between CABG and PCI with EES. Odd Ratios (OR) with 95% Confidence Intervals (CIs) were calculated and the pooled analyses were performed with RevMan 5.3 software. A total number of 5207 patients were involved in this analysis. No significant difference was observed in mortality between CABG and EES with OR: 0.90, 95% CI: 0.73–1.10; P = 0.30. Moreover, CABG was associated with a high stroke rate, with OR: 0.73, 95% CI: 0.45–1.17; P = 0.19, without any statistical significant. CABG was associated with significantly lower Major Adverse Cardiac Events and Myocardial Infarction with OR: 1.46, 95% CI: 1.05–2.04; P = 0.03 and OR: 1.46, 95% CI: 1.01–2.12; P = 0.05 respectively whereas PCI was associated with a significantly higher repeated revascularization with OR: 2.21; 95% CI: 1.76–2.77; P = 0.00001. In conclusion, significant differences were noted in several subgroups analyzing the mid-term cardiovascular outcomes between CABG and EES.

Even if Percutaneous Coronary Intervention (PCI) is a short procedure resulting in rapid discharge from the hospital when compared to Coronary Artery Bypass Surgery (CABG) which is time-consuming, more expensive and associated with late hospital discharge following surgery, the latter is associated with significantly better long-term adverse clinical outcomes, when compared with first generation Drug Eluting Stents (DES)^1^,^2^,^3^. Moreover, among the recently approved DES, several studies showed PCI with Everolimus Eluting Stents (EES) to be associated with improved clinical outcomes^4^,^5^,^6^. Nevertheless, several questions still need to be debated: if PCI with EES has shown better results compared to the other DES, what about the adverse outcomes reported when PCI with EES is compared to CABG? PCI with EES has seldom been compared to CABG through meta-analyses. Therefore, in order to know whether the mid-term adverse cardiovascular outcomes are significantly different between CABG and PCI with EES, this study aimed to compare CABG and PCI with EES in patients treated for Coronary Artery Diseases (CAD).

## Methods

### Data Sources and Search Strategy

PubMed, Medline, EMBASE and the Cochrane library were searched for studies comparing CABG and PCI with EES using the following words or phrase ‘coronary artery bypass surgery and everolimus eluting stents’. To further enhance this search, the words ‘percutaneous coronary intervention, and second generation drug eluting stents’ as well as abbreviations such as ‘CABG, PCI, and EES’ were used. References of suitable articles were also checked for relevant studies. In addition, only English language publications were considered relevant to this search strategy.

### Inclusion and Exclusion criteria

Studies were included if:

- They were Randomized Controlled Trials (RCTs) or observational studies.
- They compared CABG and PCI with EES.
- They reported adverse cardiovascular outcomes among their clinical endpoints.
- They had a mid-term follow up period above one year (more than one year but less than 5 years).

Studies were excluded if:

- They were meta-analyses, case studies or letter to editors.
- They did not compare CABG and PCI with EES.
- They did not report adverse cardiovascular outcomes as their clinical endpoints.
- They had a follow up period of less than one year.
- They were associated with the same trial (different studies involving the same trial).
- They were duplicated studies (same study obtained from different databases).

### Outcomes and follow up periods

The cardiovascular outcomes analyzed included:

- Mortality (all-cause death)
- Stroke
- Myocardial Infarction (MI)
- Repeated revascularization (including target vessel revascularization and target lesion revascularization)
- Major Adverse Cardiac Events (MACEs) consisting of death, MI and revascularization. Because only one study reported Major Adverse Cardiovascular and Cerebrovascular Events (MACCEs) consisting of death, MI, revascularization and cerebrovascular events, MACCEs were included in the subgroup analyzing MACEs.

A mid-term follow-up was defined as a follow up period longer than one year but not exceeding 5 years.

The reported outcomes with respective follow up periods have been summarized in Table 1.

### Data Extraction and Review

Five authors (PKB, MP, ART, AB and MZSS) independently assessed the eligibility of the studies considered relevant to this analysis. Information and data related to the total number of patients involved in the CABG and PCI groups respectively, the types of study involved, the patients’ enrollment period, data concerning the baseline features of the patients included, the reported cardiovascular outcomes and follow up periods were systematically extracted by the same five authors. If any of the authors disagreed about including certain studies or data, disagreements were discussed and solved among themselves. However, if a consensus could not be reached, disagreements were finally resolved and a final decision was made by the sixth author (WQH). The bias risk observed among the trials was assessed using recommendations obtained from the Cochrane Collaboration^7^. A grade A was allocated to a very low risk of bias whereas a grade E was allocated if a high risk of bias was observed.

### Statistical Analysis

The PRISMA reporting guideline was followed for this systematic review and meta-analysis^8^. Heterogeneity across the subgroups were assessed using:

- The Cochrane Q-statistic test whereby a P value less or equal to 0.05 was considered statistically significant. Any P value greater than 0.05 was considered insignificant.
- The I^2^ statistic test whereby a low value of I^2^ represented a low heterogeneity, whereas an increasing I^2^ value represented an increasing heterogeneity among the subgroups assessed.

In this analysis, if I^2^ was greater than 50%, a random effect model was used and if I^2^ was less than 50%, a fixed effect model was used appropriately. Publication bias was visually estimated by assessing funnel plots which were obtained from RevMan. Odd Ratios (OR) with 95% Confidence Intervals (CIs) were calculated for the subgroup analysis of the cardiovascular outcomes. The pooled analyses were performed/conducted with RevMan 5.3 software.

Ethical approval was not required for this type of study. All the six authors had full access to the data and approved the manuscript as written.

## Results

### Search Results

**Figure 1:**
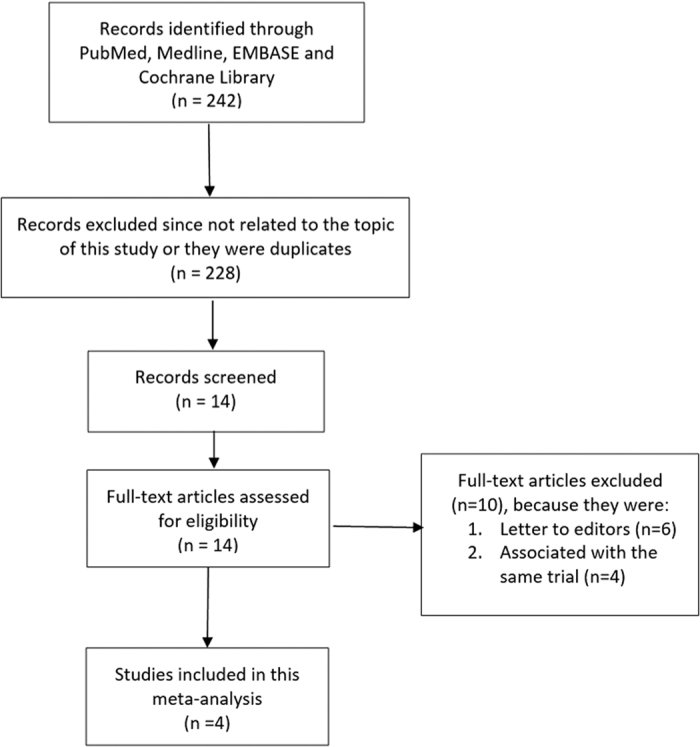
Flow diagram representing the study selection.

Two hundred and forty-two articles were obtained during the search process. After a careful assessment of the titles and abstracts, 228 articles were eliminated since they were either not related to the title of this research, or they were duplicates. Fourteen full-text articles were assessed for eligibility. A further 10 articles were eliminated since 6 articles were letter to editors whereas 4 articles were associated with the same trial. Finally, only 4 articles (2 observational studies and 2 randomized trials) which satisfied all the inclusion and exclusion criteria of this study were selected and included in this meta-analysis. The flow diagram showing the study selection has been represented in Fig. 1.

### General Features of the studies included

A total number of 5207 patients were involved in this analysis (2605 patients were treated by PCI with EES and 2602 patients were treated with CABG). The general features of the studies included in this meta-analysis have been summarized in Table 2.

For study bangalore2015, only patients who were >80 years were included. Reasons were due to the fact that all the other studies consisted of less patients and therefore, in order to match their patient number, and in order for the result of this analysis not to be influenced by the result obtained in the study Bangalore2015, only patients >80 years were considered.

### Baseline features of the studies included

The baseline features of the patients included in this study have been listed in Table 3. According to Table 3, there were no significant differences in baseline features among patients included in the EES or CABG groups.

### Analysis comparing CABG with EES

Results of this analysis have been summarized in Table 4.

**Figure 2:**
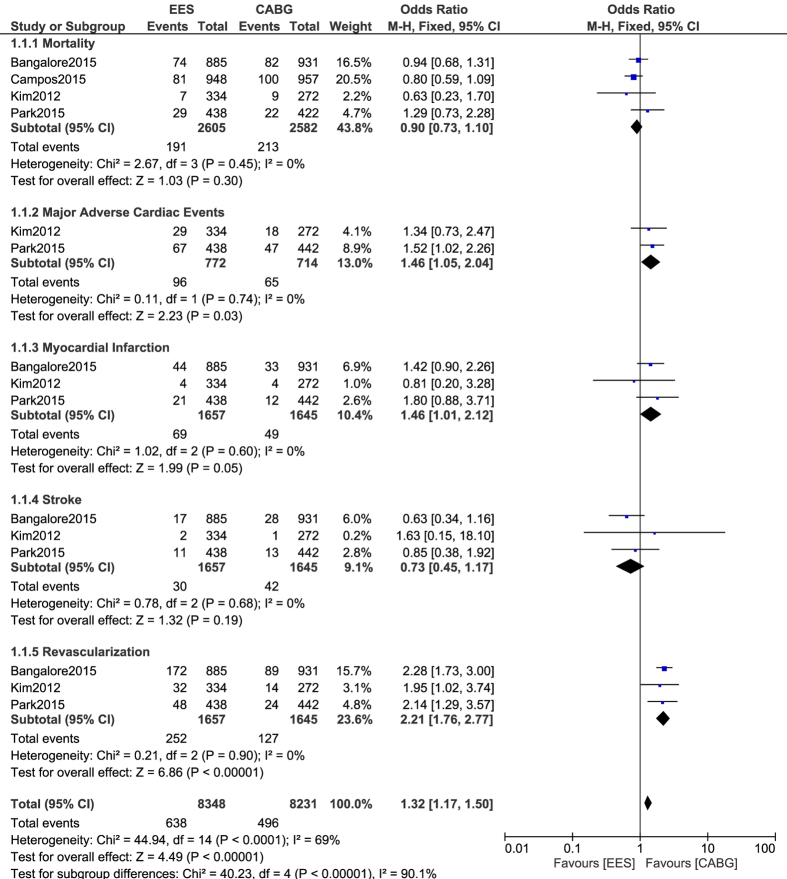
Adverse Cardiovascular Outcomes reported between CABG and PCI with EES.

This analysis showed that during a mid-term follow up period of more than one year, no significant difference was observed in mortality between CABG and EES with OR: 0.90, 95% CI: 0.73–1.10; P = 0.30, I^2^ = 0%. Moreover, CABG was associated with a higher rate of stroke, with OR: 0.73, 95% CI: 0.45–1.17; P = 0.19, I^2^ = 0%, however, the result was not statistically significant. CABG was also associated with significantly lower MACEs and MI with OR: 1.46, 95% CI: 1.05–2.04; P = 0.03, I^2^ = 0% and OR: 1.46, 95% CI: 1.01–2.12; P = 0.05, I^2^ = 0% respectively whereas PCI was associated with a significantly higher repeated revascularization with OR: 2.21; 95% CI: 1.76–2.77; P = 0.00001, I^2^ = 0%. Results showing the adverse cardiovascular outcomes between CABG and PCI with EES have been represented in Fig. 2.

**Figure 3:**
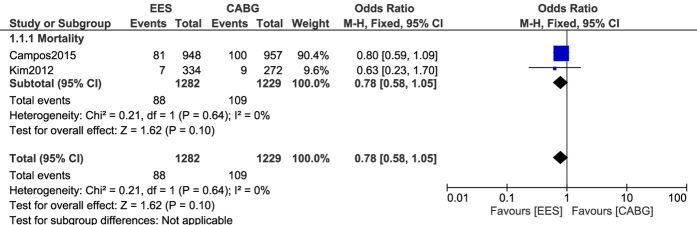
Mortality reported between CABG and PCI with EES in patients with left main coronary diseases.

Since our data included patients with multi-vessel diseases and patients with left main coronary diseases, further analyses were conducted separating these two categories of patients and comparing CABG and PCI with EES. In patients with left main coronary artery disease, mortality was also not significantly different between CABG and PCI with EES, with OR: 0.78, 95% CI: 0.58–1.05; P = 0.10, I^2^ = 0%. This result has been represented in Fig. 3.

**Figure 4:**
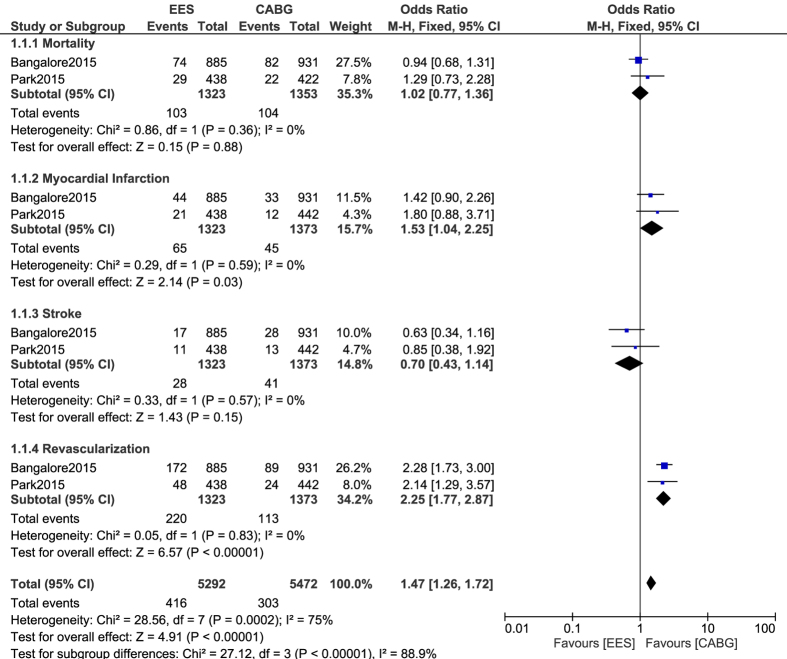
Adverse Cardiovascular Outcomes reported between CABG and PCI with EES in patients with multi-vessel coronary diseases.

In patients with multi-vessel coronary artery diseases, a similar mortality rate was observed between CABG and PCI with EES, with OR: 1.02, 95% CI: 0.77–1.36; P = 0.88, I^2^ = 0%. MI and repeated revascularization significantly favored CABG with OR: 1.53, 95% CI: 1.04–2.25; P = 0.03, I^2^ = 0% and OR: 2.25, 95% CI: 1.77–2.87; P < 0.00001, I^2^ = 0% respectively. However, even if stroke was higher in these patients with multi-vessel diseases revascularized by CABG, with OR: 0.70, 95% CI: 0.43–1.14; P = 0.15, I^2^ = 0%, the result was not statistically significant. Results showing adverse cardiovascular outcomes between CABG and PCI with EES in patients with multi-vessel coronary diseases have been represented in Fig. 4.

### Sensitivity analysis

**Figure 5:**
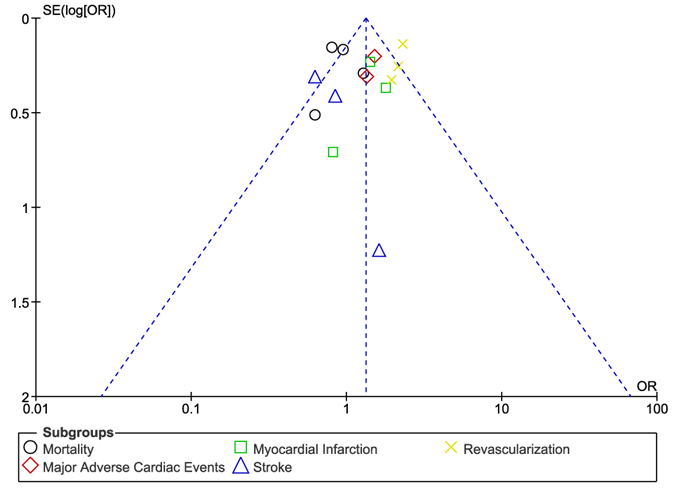
Funnel plot showing sensitivity analysis.

For the above analyses comparing CABG and PCI with EES, sensitivity analyses yielded consistent results. Based on a visual inspection of the funnel plot obtained, there has been no evidence of publication bias for the included studies that assessed all the mid-term cardiovascular outcomes. The funnel plot representing sensitivity analysis is shown in Fig. 5.

## Discussion

This study aimed to compare the adverse cardiovascular outcomes associated with CABG and PCI with EES during a follow up period of more than one year (less than 5 years). The current analysis showed no significant difference in mortality between CABG and PCI with EES. Even if a higher rate of stroke was observed with CABG, the result was not statistically significant. However, PCI with EES was associated with significantly higher MACEs, MI and repeated revascularization when compared to CABG. A similar result was obtained when patients with left main coronary artery disease and multi-vessel coronary diseases were separately analyzed.

The study published by Bangalore *et al*., including diabetic patients with multi vessel coronary diseases showed a similar mortality rate associated with EES and CABG during the long term^13^. Moreover, higher risk of MI and repeated revascularization were observed with EES, whereas a lower risk of stroke was observed with CABG. Another study by the same authors showed results similar to this current analysis whereby a similar mortality rate was observed between CABG and PCI with EES, with a higher rate of repeated revascularization associated with EES, whereas a high rate of stroke was associated with CABG. However, the study involved only patients with diabetes mellitus^14^.

The Coronary Artery Bypass Surgery and Everolimus Eluting Stent Implantation in the Treatment of Patients with Multivessel Coronary Artery Disease (BEST) trial which involved patients with multivessel diseases also showed a significantly higher rate of MACEs associated with PCI with EES when compared to CABG, at 2 years follow up 11. In addition, 11 cases of stroke were reported with PCI, whereas 13 cases were reported with CABG. The trial which involved 880 patients, was a prospective, open-labeled randomized trial conducted in several sites including Korea, China, Malaysia and Thailand. However, this current study could not analyze MACEs specifically in patients with multi-vessel diseases due to a lack of data.

In contrast, the Premier of Randomized Comparison of Bypass Surgery versus Angioplasty Using Sirolimus-Eluting Stent in Patients with Left Main Coronary Artery Disease (PRECOMBAT 2)^10^ study showed EES to be associated with a similar MACCEs when compared to CABG at 18 months follow up. However, only patients with upper left main coronary artery stenosis were involved. This current analysis combined MACEs and MACCEs together and showed a significantly higher rate of MACEs in the EES group. When patients with left main coronary diseases were separately analyzed in this current research, mortality was not significantly different between these two revascularization procedures.

At last, this current analysis satisfied all the criteria for a meta-analysis in terms of low level of heterogeneity, low risk of bias, and robust results. Therefore, it might be used by physicians and interventionists in order to select treatment strategies in patients with CAD, and to predict mid-term prognosis. However, further studies with long term follow up period should be recommended to further debate on this very interesting issue particularly relevant to clinical medicine.

### Novelty

This research is new in the field of interventional cardiology. Only a few trials or cohort studies have compared CABG and PCI with EES. However, this is currently the first meta-analysis combining data obtained from the few published studies to conduct a new analysis showing whether similar or different outcomes were reported between CABG and PCI with EES, representing a new idea in clinical medicine. In addition, a very low level of heterogeneity obtained among all the subgroups analyzed, might represent another new feature of this study. Moreover, physicians and interventionists might be able to predict prognosis or select revascularization strategies in patients with CAD, taking into consideration the cost as well as other benefits or disadvantages of these respective procedures.

### Limitations

Limitations in this analysis were as follows: A limited number of patients were analyzed. Lack of data might have had an effect on the result of this analysis. The study Bangalore2015 consisted of more than 15,000 patients. However, only patients above 80 years old were extracted from that study to be included in this current analysis. Reasons for extracting only patients above 80 years old were to match the number of patients obtained from the other studies in order to obtain a smooth result which would not be based only on the dominant study Bangalore2015. Moreover, similar endpoints were not reported in all the studies included in this analysis restricting the comparison of several outcomes, showing another limitation of this analysis. In addition, the fact that patients with diabetes mellitus, multi-vessel coronary diseases and left main coronary artery diseases were combined and analyzed could represent another limitation of this study since this combination of patients with different co-morbidities could also have had a major impact on the results.

## Conclusion

Significant differences were noted in several subgroups analyzing the mid-term cardiovascular outcomes associated with CABG and PCI with EES. Mortality and stroke were not significantly different between these two revascularization procedures. However, PCI with EES was associated with significantly higher MACEs, MI and repeated revascularization when compared to CABG. Unfortunately, due to limited data, which could have affected the results of this analysis, further studies with larger population size and longer follow up periods are recommended to completely solve this issue.

## Additional Information

**How to cite this article**: Bundhun, P. K. *et al*. Adverse Cardiovascular Outcomes associated with Coronary Artery Bypass Surgery and Percutaneous Coronary Intervention with Everolimus Eluting Stents: A Meta-Analysis. *Sci. Rep.* **6**, 35869; doi: 10.1038/srep35869 (2016).

## Figures and Tables

**Table 1:** Reported outcomes with respective follow up periods.

Studies	Reported outcomes	Follow up periods
Bangalore2015	Death, MI, stroke, revascularization	2.9 years
Kim2012	Death, MI, stroke, revascularization, MACCEs	1.5 years
Park2015	Death, MI. stroke, revascularization, MACEs	2 years
Campos2015	Mortality	4 years

Abbreviations: MI: myocardial infarction, MACEs: major adverse cardiac events, MACCEs: major adverse cardiovascular and cerebrovascular events.

**Table 2:** General features of the studies included.

Studies	Patients’ enrollment	Type of study	No of patients in EES group (n)	No of patients in CABG group (n)	Total no of patients (n)	Bias score
Bangalore2015^9^	2008–2011	observational	885	931	1816	—
Kim2012^10^	2009–2010	observational	334	272	606	—
Park2015^11^	—	RCT	438	442	880	B
Campos2015^12^	2010–2014	RCT	948	957	1905	B
Total no of patients (n)			2605	2602	5207	

Abbreviations: RCT: randomized controlled trials, EES: everolimus eluting stents, CABG: coronary artery bypass surgery.

**Table 3:** Baseline features.

Studies	Mean age (y)	Males (%)	Hypertension (%)	Dyslipidemia (%)	DM (%)
	**EES/CABG**	**EES/CABG**	**EES/CABG**	**EES/CABG**	**EES/CABG**
Bangalore2015	65.1/65.1	72.6/72.9	—	—	39.0/39.5
Kim2012	62.9/62.5	70.7/76.8	56.6/51.5	44.6/39.3	34.7/30.1
Park2015	64.0/64.9	69.4/73.5	67.6/66.7	54.6/50.2	40.4/42.1
Campos2015	66.0/66.0	76.2/77.6	—	—	—

Abbreviations: EES: everolimus eluting stents, CABG: coronary artery bypass surgery, DM: diabetes mellitus, y: years.

**Table 4:** Results of this analysis.

Outcomes analyzed	OR with 95% CI	P value	I^2^ (%)
Mortality	0.90 [0.73–1.10]	0.30	0
Myocardial Infarction	1.46 [1.01–2.12]	0.05	0
Major adverse cardiac events	1.46 [1.05–2.04]	0.03	0
Stroke	0.73 [0.45–1.17]	0.19	0
Repeated revascularization	2.21 [1.76–2.77]	0.0001	0

Abbreviations: OR: odds ratios, CI: confidence intervals.
